# Odometry for Ground Moving Agents by Optic Flow Recorded with Optical Mouse Chips

**DOI:** 10.3390/s141121045

**Published:** 2014-11-06

**Authors:** Hansjürgen Dahmen, Hanspeter A. Mallot

**Affiliations:** Cognitive Neuroscience, University of Tübingen, Auf der Morgenstelle 28, Tübingen 72076, Germany; E-Mail: hanspeter.mallot@uni-tuebingen.de

**Keywords:** odometry, optic flow, multiple optical mouse sensors

## Abstract

Optical mouse chips—equipped with adequate lenses—can serve as small, light, precise, fast, and cheap motion sensors monitoring optic flow induced by self motion of an agent in a contrasted environment. We present a device that extracts self motion parameters exclusively from flow in eight mouse sensors. Four pairs of sensors with opposite azimuth are mounted on a sensor head, each individual sensor looking down with −45° elevation. The head is mounted on a carriage and is moved at constant height above a textured planar ground. The calibration procedure and tests on the precision of self motion estimates are reported.

## 1. Introduction

Optic flow (OF) is used by many insects for flight control [1,2]. Bees control landing, flight speed, object and travelling distances by OF (see the comprehensive review by Srinivasan [3]). There is a vast literature on the influence of OF on the control of flight speed, chasing behavior and turning responses in various species of flies (see the review by Egelhaaf [4]). The present work was inspired by findings on the visually controlled behavior of water striders (*Gerris lacustris, Gerris paludum*) [5–7]. These animals efficiently compensate for body rotation by head counter-rotation based on OF information provided by a visual system with panoramic field of view and low spatial resolution. This observation raises the question of how to distribute a limited number of small-field motion sensors over the view sphere in order to optimally estimate self-motion from OF. Precision limits for the estimation of self-motion parameters from OF in a static surround were investigated under various environmental conditions using different parts of the visual field [8,9]. In these studies, self-motion from OF was determined using the algorithm proposed by Koenderink & van Doorn [10] for spherical field of view eyes. The result was that self-motion can be extracted from flow to a surprisingly high precision if flow can be observed by detector pairs with opposite viewing directions and if these pairs are distributed over a large solid angle. Under these conditions, a small number of properly combined flow measurements is sufficient for good self-motion estimates, provided that the environment is static and detectable contrasts are abundant. The extraction of self-motion from OF in nine viewing directions distributed over the entire visual sphere and using suitably oriented Reichardt motion detectors was modeled by Neumann and Bülthoff [11]. In flies, “large field neurons” have been found with motion response fields, *i.e.*, patterns of locally preferred motion directions, reflecting global flow fields of particular rotatory or translatory self-motions. A mathematical model of self-motion estimation via matched filters of this type has been suggested by [12,13].

In addition to the interest in the exploitation of OF in animals there is a lot of work in robotics devoted to the problems of structure from motion, obstacle avoidance, and self-motion control (for a review see [14,15]). To extract self-motion from flow, Baker *et al.* [16] and Pless [17] showed that an omni-directional view helps a lot to eliminate ambiguities in the evaluation of self-rotation and -translation in the case both are present. A number of catadioptric systems have been developed in order to realize omni-directional vision with a single camera [18–22]. Recently, an attempt has been made to engineer an artificial compound eye with a half-spherical field of view (FOV) [23]. The approach most similar to ours is the so called argus eye [16,24]. In this system several cameras with non-overlapping visual fields looking into various—preferably opposite—directions cooperate to reveal self-motion parameters and the structure of the environment. Besides the difficulty of calibrating such a system, one has to deal with the integration of the output of several cameras [17]. In this paper, we present a much simpler and faster setup, monitoring OF by commercially available dedicated flow detectors.

With the development of optical mouse chips (OMC), cheap (2.5 Euro), light (0.5 g), and fast (response time <1 ms) mass- produced flow detectors have become available. These sensors can be adapted for OF detection by attaching a suitable lens in front of the light-sensitive area in order to image the optical environment to the sensor. In addition, multiple such sensors can be read out simultaneously via a microprocessor. This design was first used and tested in an omni-directional treadmill (air supported ball in socket) developed for investigating the behavior of rats in a virtual environment [25]. Two OMCs (HDNS2000 Agilent) were used as motion detectors and equipped with small, high quality plastic lenses (CAY046 Philips). The microprocessor extracted the displacement of the ball surface images fast and simultaneously on the two OMCs. With this set-up, rotations of the ball about all three axes could be registered. OMCs can also be used as OF detectors in general, contrasted environments by simply adjusting the lens distance according to the distance of the imaged scene. Using a focal length of 4.6 mm, the image quality is sufficiently good for distances from 10 cm to infinity.

Odometry and its precision limits with several arrangements of mouse chips on ground moving robots have been investigated by [26–30]. In all of these studies the sensors look down to the ground vertically. In some of them [28–30] lenses different from those applied in computer mouse applications are attached to the sensors in order to allow for larger distances to the ground, thus reducing errors caused by distance variations. In the present paper, we use the combined recordings of eight sensors looking down at −45° of elevation with a distance of about 15 cm to the ground. This is a compromise between the best orientation of sensors in order to register rotation of the robot around the vertical axis (best orientation of sensors 0° of elevation) and translation on the ground (best sensor orientation −90° of elevation). The integration of the sensors' output is motivated by the matched filter approach found in the flies' visual system and explained in Section 3.

Here, we present a hardware realization of an odometer driven solely by simultaneous flow measurements by mouse sensor chips along eight lines of sight in space.

A preliminary report of an OMC-based odometer was presented at the conference ‘Flying Insects and Robots’ held in Switzerland in 2007 [31]. Similar ideas have been applied to obstacle avoidance in unmanned arial vehicles [15,32].

## 2. Hardware Implementations

**Figure 1.**
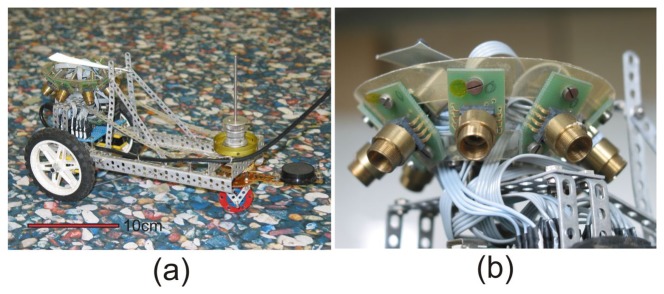
(**a**) Three wheel carriage with sensor head on flat ground (textured with the image of pebbles); (**b**) The sensor head in detail above the hind wheels. The sensor head contains eight optical mouse sensors (ADNS2620, Avago) looking at 45° relative to each other in azimuth and about −45° relative to the horizon down to the ground. Each sensor is equipped with a distance-adjustable plastic collimator lens (CAY046 Philips) of f = 4.6 mm focal length which images the floor onto the light sensitive area of the sensor. On top of the head an elongated triangle of reflex foil is attached as a target for tracking by a video camera looking down from the ceiling.

**Figure 2.**
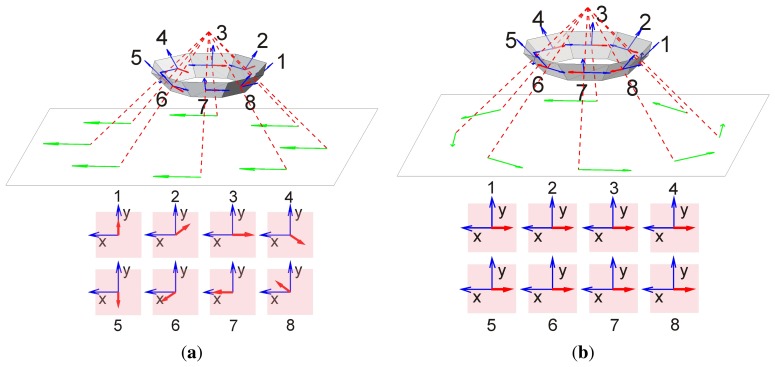
Sensor configuration and expected flow pattern. Each sensor marked with the sensor number has a viewing direction shown as a red dashed line and an image plane indicated by a gray transparent trapezoid. The directions of *X*- and *Y* -components in each image plane are indicated by blue arrows. The *X* direction is in all sensors nearly parallel to the horizontal plane. The red arrows show the optic flow induced in the sensors' image planes by the ground velocity indicated by green arrows. The upper figures in (**a**) and (**b**) show a view onto the eight-sensors head from above and the side, the lower part shows the eight light sensitive areas and the corresponding optic flow. Figure 2a illustrates pure translation in forward direction (*i.e.*, aligned with sensor 1), Figure 2b pure clockwise rotation (yaw).

The prototype of a sensor head with eight sensors has been mounted on a small three wheel carriage (Figure 1). It is designed for ground moving robots for which the distance to ground does not change too much. The viewing directions of the eight sensors are oriented at angular distances of 45° to each other in azimuth and look down to the ground at −45° below the horizon (Figure 2). In this paper, we consider vehicles with two degrees of freedom (DOF) of self-motion, *i.e.*, yaw and forwards-backwards translation, but extensions to general movements in the plane are straight-forward.

On top of the sensor head, an elongated triangle of reflex foil (3M Scotchlite type 7610) is attached which allows to track the position and the angular orientation of the head by a video camera looking down from the ceiling. The reflex marker moves in a horizontal plane 7 cm above the ground.

Each of the eight mouse sensors ADNS2620 (Agilent) samples the light intensity pattern on its 1×1 mm array of 18 × 18 light sensitive diodes (LSD) with a rate of about 1500 frames/s. The focal length of the lens (f = 4.6 mm) and the size of the sensor diode array determine the FOV of the sensors to 12.4° × 12.4°. A fast on-chip digital signal processor (DSP) calculates the displacement between two consecutive light intensity patterns using a correlation technique. In order to avoid too large displacements between subsequent images the maximum allowed speed of the pattern on the chip's light sensitive surface is limited to *υ_max_* = 300 mm/s. This limits the range of the detectable rotation speed (about an axis perpendicular to the optical axis) to a maximum of

(1) $$\frac{\upsilon_{\max}}{f}\times \frac{180{}^{\circ}}{\pi }=3737{}^{\circ}/s$$

With a viewing distance **D**[cm] to ground the range of detectable translational speeds is limited to a maximum of

(2) $$(300/f)\text{D}=65{.22s}^{-\text{1}}\times \text{D}$$

(*i.e.*, about 980 [cm/s] for D = 15 cm). The minimal detectable displacement is specified to 1/400 inches = 1/16 mm. With the focal length of 4.6 mm this leads to an angular resolution of 0.775° and to a minimum detectable displacement of 2.04 mm at a distance of 15 cm.

The proper adjustment of the lens distance can be controlled by running the OMCs in the very slow so called ‘Pixel Data’ mode. In this mode the actual image of a still scene on the pixel surface of the chip can be displayed in order to control image quality.

**Figure 3.**
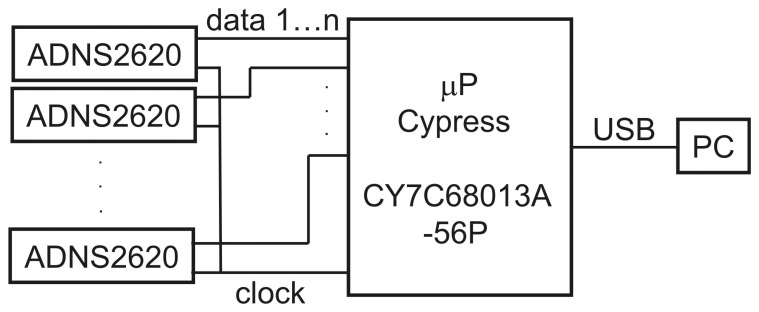
Circuit diagram of the odometer. A microprocessor (*μ*P) (CY7C68013A-56P, Cypress) reads information synchronously from all sensors in parallel via two serial lines to each sensor : a clock- and a data line. The clock line is common to all sensors and guarantees synchronous data transfer to and from all sensors. The data line of each sensor is connected to an individual I/O pin on the *μ*P. The *μ*P is connected to a PC via USB.

A microprocessor (*μ*P) (CY7C68013A-56P, Cypress) reads information from all sensors at a rate of about 140 Hz in parallel and synchronously (Figure 3). The information consists of three bytes: *dY*, *dX*, *SQ*, in that order. *dY*, *dX* are the pattern displacements since the last reading measured along each sensor's (*Y*, *X*) axes, *SQ* is a ‘quality’ byte. If *SQ* falls below a selectable threshold, *dY* and *dX* may be unreliable and are discarded. Reading the information from all sensors (strictly synchronously in parallel) and transferring them via an USB1.1 bulk transfer to the PC takes less than 2 ms.

**Figure 4.**
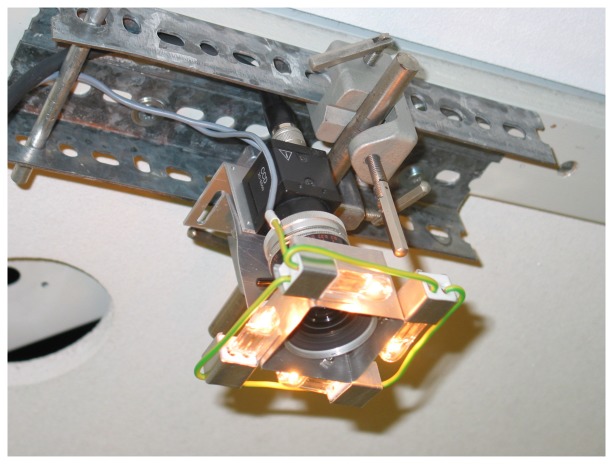
The sensor head is tracked by a compact analog video camera (Sony CCD XC-ES50) mounted at the ceiling of the lab 2.7 m above the reflex foil triangle plane on top of the sensor head. The attached high quality lens (Kern Macro-Switar, f = 10 mm) covers a FOV of 165 × 125 cm^2^ on the ground. The lens is surrounded tightly by four halogen bulbs the brightness of which can be adjusted. Thus the image of the reflex foil triangle can be discriminated by its brightness and high contrast against the rest of the environment.

Angular orientation and position in space of the sensor head are tracked by a video analogue compact CCD camera (Sony CCD XC-ES50) equipped with a C-mount high quality lens (Kern, Macro-Switar, f = 10 mm, FOV 26° × 34°) mounted 2.7 m above the head's reflex marker plane. The observed area on the ground was 165 × 125 cm^2^. The lens is surrounded as tightly as possible by four halogen bulbs, the brightness of which can be adjusted. A high fraction of the light of these bulbs is reflected back into the lens by the reflex foil, while the amount of light reflected by the rest of the environment is much smaller. Thus the triangle stands out with high contrast against the rest of the vehicle and the ground and can be detected by thresholding. The camera is connected to the PC via a standard frame grabber board (640 × 480 pixel, 50 frames/s), see (Figure 4).

## 3. Calibration

### 3.1. Calibration of Head Translation and Rotation

The path of the carriage is a planar curve which can be approximated at any given moment by a horizontal circle. This means that at any moment the motion of the carriage can uniquely be decomposed into a translational component along its long axis and a yaw component about the center of the head (see Figure 2). The response to pure forwards translation and yaw is illustrated by Figure 2a,b, respectively. Sensor 1 looks in the forwards direction.

**Figure 5.**
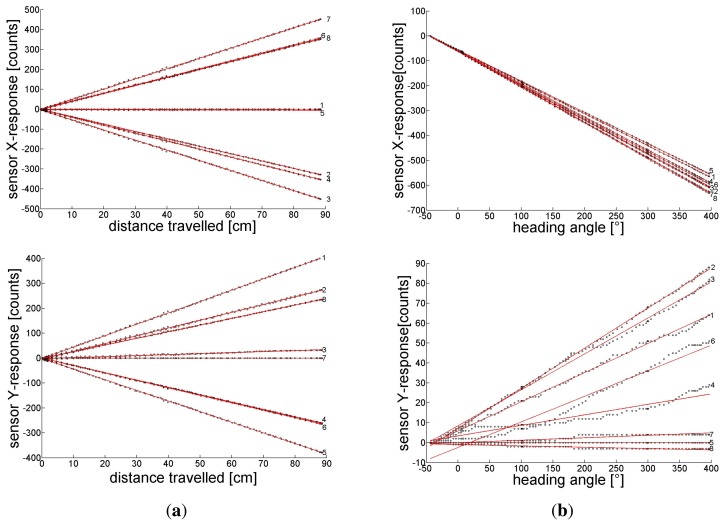
The accumulated X- (upper) and Y-response (lower row of sub figures) of the eight sensors to pure translation (column a) and pure yaw (column b) versus the traveled distance (**a**) and the orientation of the carriage (**b**) monitored by the tracking camera. Every fifth sensor response is marked by a black dot, red lines are a linear fit to them. The slopes of the linear fits in column (a) represent the (*X*, *Y*) ‘unit’ responses ${\left(a_{x}^{i},a_{y}^{i}\right)}^{⊤}$ of the sensors to 1 cm of translation and those in column (b) to 1° of rotation ${\left(b_{x}^{i},b_{y}^{i}\right)}^{⊤}$. For further discussion of the responses see text.

In order to use the head for odometry, we calibrate the sensitivity of each individual sensor to (a) forwards translation and (b) yaw rotation. For this purpose the orientation of the front wheel is fixed (a) in parallel or (b) orthogonal to the long axis of the carriage. The carriage is pushed by hand and moves either in case (a) along a straight line without rotation or rotates in case (b) around the center of the sensor head without translation. The position and orientation of the carriage are monitored by the tracking camera and the output of all sensors is registered. The ground plane was textured with an image of pebbles (see Figure 1). Sensor responses are linear in the range of at least 1.25°/s to 1500°/s [31]. Frame-by-frame flow measurements can therefore be integrated over extended motion sequences to obtain more accurate results. The upper two sub figures in Figure 5 show the *X* response of each sensor, the two lower ones the *Y* response to the accumulated flow induced by a pure forwards translation (sub figure column a) and by a pure yaw to the right (sub figure column b), respectively. The sensor number is indicated at the right margin of each sub figure.

‘Unit’ responses to translations of 1 cm and rotations of 1° are given by the slope of the accumulated sensor readings which were fitted by linear regression. The slope of this regression in column (a) is taken as the (*X*, *Y*) unit response to 1 cm of translation, ${\text{a}}^{i}={\left(a_{x}^{i},a_{y}^{i}\right)}^{⊤}$ where *i* denotes the sensor number and ^⊤^ marks transposition. Similarly, the slope in column (b) is taken as the (*X*, *Y*) unit response to 1° of yaw, ${\text{b}}^{i}={\left(b_{x}^{i},b_{y}^{i}\right)}^{⊤}$.

The responses shown in Figure 5, column a and b, reflect the sensor arrangement, depicted in Figure 2a,b, respectively. The pattern of responses is characteristic for each DOF: in sensors with opposite azimuth (e.g., sensor pairs 1–5, 2–6, *etc.*) translation induces flow in opposite (*X*, *Y*)-direction whereas yaw induces in all sensors approximately the same flow, mainly in X-direction. These response patterns depicted in the lower panel of Figure 2a,b can be used as templates or matched filters which allow the extraction of each self-motion component in the case of superimposed translation and yaw (see Equations (6) and (7) in Section 4). The response patterns to translation and rotation of sensor pairs looking in opposite directions (*i.e.*, 1–5, 2–6, *etc.*) are approximately orthogonal to each other.

**Figure 6.**
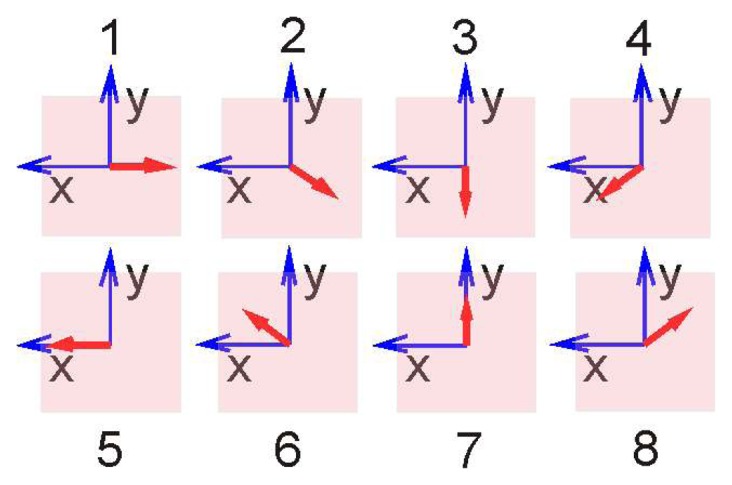
Sensors' light sensitive area and expected flow pattern for sideways translation analogous to lower Figure 2a,b.

Note that our head is also capable of measuring sideways translation as another DOF. This DOF can be extracted by a third matched filter illustrated in Figure 6 orthogonal to that of Figure 2a. We do not pursue this idea further but show mathematics and experiments for the two DOF case.

The response $\text{c}={\left(c_{x}^{1},c_{y}^{1},\ldots ,c_{x}^{n},c_{y}^{n}\right)}^{⊤}$ of *n* sensors to a combined motion of *τ* cm of translation and *ρ* degrees of yaw can be written as

(3) c=Um

where **m** = (*τ*, *ρ*)^⊤^ and **U** = (**a**, **b**), the unit response matrix, consists of two columns, the first $\text{a}={\left(a_{x}^{1},a_{y}^{1},\ldots ,a_{x}^{n},a_{y}^{n}\right)}^{⊤}$ representing the unit response of the *n* sensors to translation and $\text{b}={\left(b_{x}^{1},b_{y}^{1},\ldots ,b_{x}^{n},b_{y}^{n}\right)}^{⊤}$ that to yaw, respectively.

### 3.2. Calibration of the Tracking Camera

For the calibration of the tracking camera (Figure 4) a horizontal disk of reflex foil of 3.3 cm diameter was successively positioned at the nodes of a rectangular grid of six by five lines laid out on the ground with a spacing of 25 cm in *X* and *Y* direction. The height of the reflex foil disk above ground was identical to that of the carriage's reflex triangle. The coordinates and brightness of the video pixels imaging the reflex disk were extracted using OpenCV. From the pixels passing an intensity threshold, the intensity-weighted center of gravity (CG) was calculated and assigned to the corresponding ground coordinates. The (*X*, *Y*) coordinates of the ground plane were aligned with the (*x*, *y*) coordinates of the camera. The optical axis of the camera was adjusted orthogonally to the ground plane so that no trapezoidal distortion of the image was visible. The quality of the lens resulted in no barrel-shaped image deformation visible within the error limits of the determination of the CG. Thus the pixel coordinates (*x, y*) of the tracking camera were assigned to the (*X*, *Y*) coordinates of the ground system using a simple camera model.

(4) $$\begin{array}{ll} x-x_{0}=s_{x}X; & y-y_{0}=s_{y}Y \end{array}$$

where (*x*_0_, *y*_0_) = (148.1962 ± 0.2776 , 68.2872 ± 0.3296) denote the origin of the ground coordinate system in camera coordinates and (*s_x_*, *s_y_*) = (3.5910 ± 0.0037, 3.9145 ± 0.0053) are (*x*, *y*) scaling factors including focal length, object distance, and pixel size. *x*_0_, *s_x_* and *y*_0_, *s_y_* were evaluated by a linear fit over all *x*- versus *X*-coordinates and *y*- versus *Y* -coordinates, respectively. The standard deviation in reconstructing the ground coordinates (*X*, *Y*) from image coordinates (*x*, *y*) were (0.86 cm, 1.03 cm). Obviously the pixels were not quadratic but had a slightly larger extension in x-direction.

## 4. Odometry

As long as the distance of the sensors to the ground is constant, we can use the stream of sensor responses **c***^i^* and Equation (3) to extract the displacement *τ* and the rotation *ρ* between two sensor responses. In order to find the best solution for **m** we have to solve Equation (3) as

(5) $${\left({\text{U}}^{⊤}\text{U}\right)}^{-1}{\text{U}}^{⊤}\text{c}=\text{m}$$

With U^⊤^U = (a^2^, ab; ab, b^2^) and (U^⊤^U) ^−1^ = (b^2^, −ab; −ab, a^2^)/det(U^⊤^U), the solution of Equation (5) reads

(6) $$\tau =({\text{b}}^{2}(\text{ac})-(\text{ab})(\text{bc}))/D$$

(7) $$\rho =({\text{a}}^{2}(\text{bc})-(\text{ab})(\text{ac}))/D$$

with

(8) $$\text{D}=\det({\text{U}}^{⊤}\text{U})={\text{a}}^{2}{\text{b}}^{2}-{\left(\text{ab}\right)}^{2}$$

The new orientation *α_t_*_+1_ and the new coordinates X*_t_*_+1_, *Y_t_*_+1_ are then iterated from the previous *α_t_*, *X_t_*, *Y_t_* by a trapezoidal rule:

(9) $$\begin{array}{lll} \alpha_{t+1} & = & \alpha_{t}+0.5\;(\rho_{t}+\rho_{t+1})\\ X_{t+1} & = & X_{t}+0.5\;(\tau_{t}\cos\alpha_{t}+\tau_{t+1}\cos\alpha_{t+1})\\ Y_{t+1} & = & Y_{t}+0.5\;(\tau_{t}\sin\alpha_{t}+\tau_{t+1}\sin\alpha_{t+1}) \end{array}$$

The odometer can only be expected to monitor the *increments τ* of position and *ρ* of orientation over time, so (*X*_0_*, Y*_0_) and *α*_0_ at time t = 0 must be known from elsewhere.

## 5. Tests

In order to get a first estimate of the precision of odometer results from our sensor head it was moved manually 20 times along a straight line for distances *τ* of 20, 40, 60, and 80 cm and rotated 20 times around the vertical axis by *ρ* = 90°, 180°, 270°, and 360°. We take these values as ground truth, ignoring possible errors of the manually performed movements. Averages and standard deviations of estimates *τ̄* and *ρ̄* for these trials are given in Table 1.

For a more rigorous test of the performance of the sensor head, we moved it on a flat ground textured with the pebbles image (see Figure 1) and recorded the position (*x_c_*, *y_c_*) and the orientation *α* of the carriage *i.e.*, of the reflective triangle on top of the sensor head by the video camera at the ceiling (see Section 2). Pixel-coordinates (*x_i_*, *y_i_*)and -brightnesses (*h_i_*) were extracted using OpenCV. Coordinates (*x_c_*, *y_c_*) of the carriage are taken as the center of gravity (CG):

(10) $$\begin{array}{ll} x_{c}=\sum h_{i}\;x_{i}/\sum h_{i}; & y_{c}=\sum h_{i}\;y_{i}/\sum h_{i}; \end{array}$$

where the sum is taken only over points (*x_i_*, *y_i_*) the image brightness *h_i_* of which is above a safe threshold to discriminate the reflective triangle from any background.

From the image coordinates (*x_c_*, *y_c_*), world coordinates (*X_c_*, *Y_c_*) were calculated according to Equation (4). The best estimate of the orientation *α* of a body consisting of points P*_i_* with coordinates (*X_i_*, *Y_i_*) relative to the center of gravity (CG) and weight *h_i_* can be found by evaluating the orientation of the intensity weighted principal axis,

(11) $$\alpha =0.5\arctan\;[2\sum h_{i}X_{i}Y_{i}/\sum h_{i}\;(x_{i}^{2}-Y_{i}^{2})]$$

**Figure 7.**
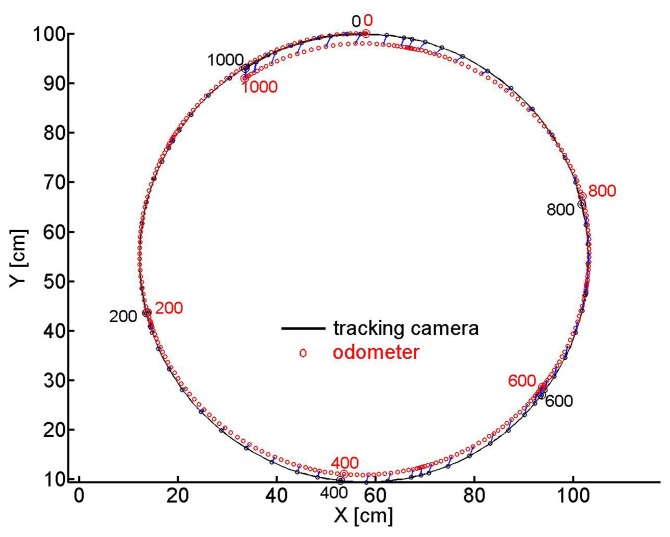
(*X*, *Y*) coordinates of the circular track of the odometer pushed by hand. The black curve shows the trace by the tracking camera. Every 2nd odometer sample is marked by a red ○. Every 8th pair of samples is connected by a blue line to illustrate the error between corresponding camera- and odometer coordinates. Every 200th sample is marked by a bigger ○ and its sample number, black for the camera and red for the odometer.

Several tracks of the odometer carriage were recorded, we present two of them. We fixed the angle of the front wheel relative to the long axis of the carriage and pushed the latter by hand so that it ran along a circle the (*X*, *Y*) coordinates of which are shown in Figure 7. The track taken by the camera is depicted by the black line and every 8th sample of coordinates is marked by a black circle. A red circle marks every 2nd *X*, *Y* -sample evaluated from the odometer recordings. Every 8th sample of coordinate pairs is connected by a blue line to illustrate the error between tracking camera and odometer recordings. Every 200th sample is marked by the sample number and a bigger circle (black for the camera and red for the odometer). At time 0, tracked and estimated positions and angles are assumed to be equal.

**Figure 8.**
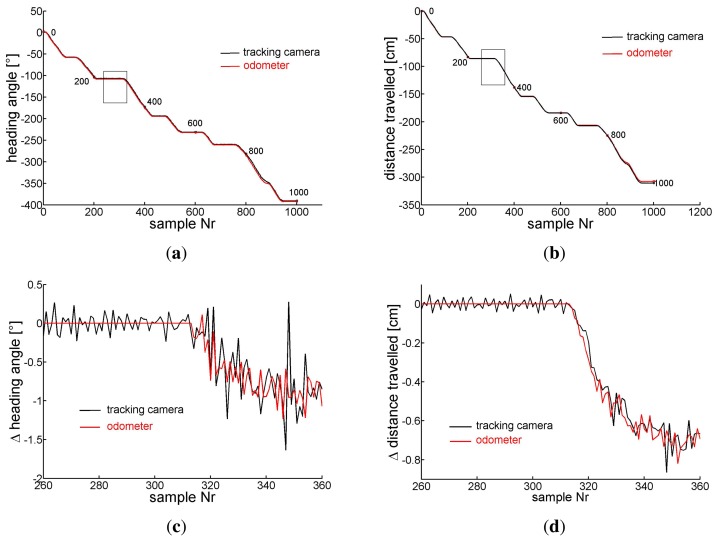
(**a**) The corresponding entities, the accumulated *ρ* (red) (Equation (7)) and the orientation (black) of the camera image (Equation (11)). (**b**) The accumulated *τ* (Equation (6)) (red) and the travelled distance of the carriage taken by the camera (black) on the circle path of Figure 7. (**c**) *ρ* (red) and the change of the orientation of the carriage (black) between two samples, (**d**) *τ* and travelled distance between two samples of camera images. (c) and (d) are taken in the time intervals indicated by the rectangles in sub figures (a) and (b), respectively.

Figure 8 shows for the same experiment versus the number of samples in (a) the orientation of the carriage extracted from the camera images (Equation (11)) (black) and the accumulated *ρ* evaluated from the odometer recordings (red) (Equation (7)), in (c) *ρ* (red) and the changes of the carriage orientation between two samples seen by the camera (black)) in the sample interval indicated by the rectangle in (a). Similarly in (b) the travelled distance taken from the camera *X*, *Y* coordinates (Equation (10)) (black) and the accumulated *τ* gained by odometry (Equation (6)) (red). In (d) the differential camera path length (black) and *τ* (red) between two samples are depicted in the sample interval indicated by the rectangle in sub figure (b). Horizontal pieces of the traces indicate standstill of the carriage between hand pushes. Note the different scales.

**Figure 9.**
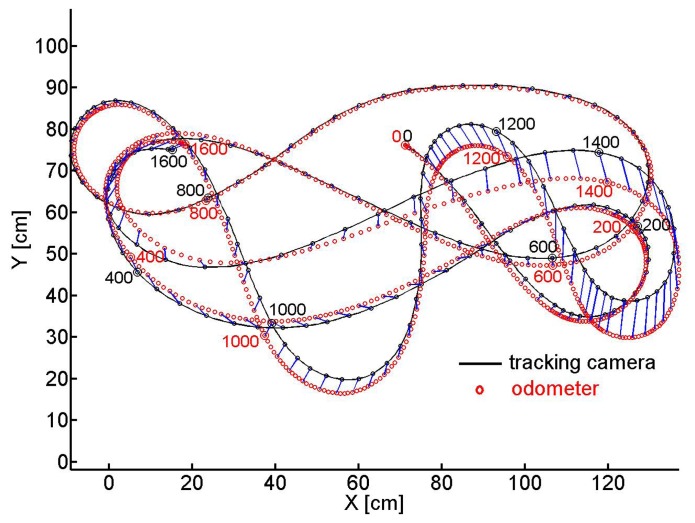
(*X*, *Y*) coordinates of the 10m curved track of the odometer dragged by hand. The data are presented in a similar way as in Figure 7.

**Figure 10.**
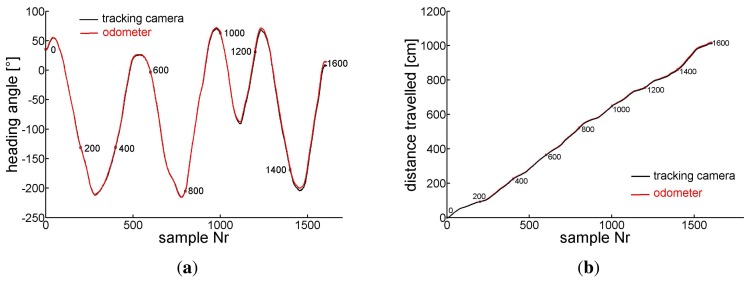
(**a**) The accumulated *ρ* obtained from the odometer (red) (Equation (7)) and the angular orientation of the carriage extracted from the camera recordings (black) (Equation (11)) during the track shown in Figure 9 are plotted versus the sample number. (**b**) The accumulated *τ* (red) (Equation (6)) and the distance travelled on the curved track are plotted versus the sample number.

In a second test we dragged the carriage along a curved path of about 10 m length. The *X*, *Y* coordinates are depicted in Figure 9. As in Figure 7 the black trace represents the track of the camera (Equation (10)) while the red circles represent the carriage's positions that are calculated from the integrated sensor responses (*τ*, *ρ*) of the odometer (Equations (6) and (7)). Again, blue connection lines between every 8th pair of coordinates indicate the deviation between the position estimates of the tracking camera and the odometer. Similarly to sub Figure 8c,d we show in Figure 10a,b the angular orientation and the traveled distance, respectively, versus the sample number.

**Figure 11.**
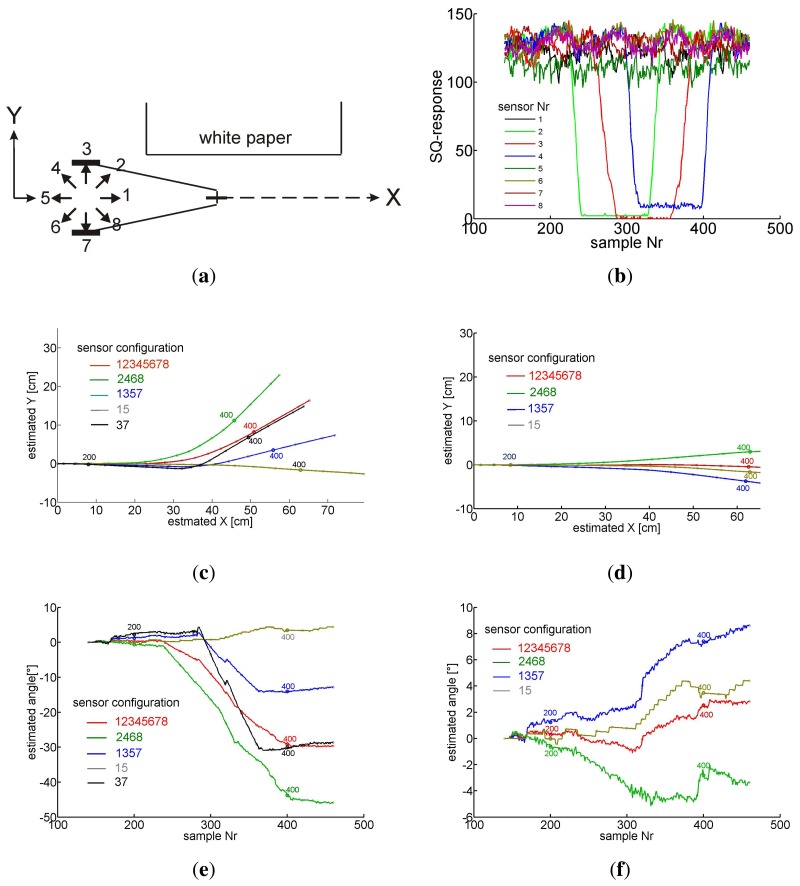
(**a**) the carriage is pushed along a straight line with a blank sheet of white paper on its left side. Eight numbered arrows indicate the azimuth of the eight mouse sensors; (**b**) The *SQ*-responses of the sensors are plotted versus the sample number. Only sensors 2, 3, 4 look to the blank zone, one after the other, while their *SQ*-response drops to low numbers; (**c**) The estimated *X*, *Y* -position of the vehicle using the responses of different sensor combinations, neglecting their *SQ*-responses (**d**) estimated *X*, *Y* -position of the vehicle using for each frame only sensors with *SQ*-response larger than the threshold of 90 (**e**) the evaluated angle of the vehicle's path when the *SQ*-responses are neglected and (**f**) when for each frame only sensors with *SQ*-response larger than 90 are used. In (c), (d), (e), (f) every 20th sample is marked by a point and every 200th sample by a number. Note the scales.

In a third test we pushed the carriage along a straight path of about 60 cm passing an area of low contrast on one side of the vehicle, see Figure 11a. A blank sheet of white paper was positioned to the left side of the path so that only sensors 2, 3, 4 looked to the paper with some time delay between them. The rest of the ground plane was covered with the usual contrasted pattern. The *SQ*-response reflects the pattern contrast on the sensor's image plane (see Figure 11b). The track of the carriage was evaluated by odometry (see Section 4) taking into account various combinations of sensor pairs, indicated in the number insets of Figure 11c–f. In Figure 11c,e the *dX, dY* -responses of the sensors were taken into account irrespective of their *SQ*-response, whereas in Figure 11d,f their response was suppressed and the corresponding rows of U (Equation (3)) were deleted as soon as the *SQ*-response dropped below the threshold of 90. Comparing Figure 11c,e to Figure 11d,f the marked improvement of odometry by taking the *SQ*-responses of the sensors into account is obvious. The results for various combinations of sensor pairs show that in the case of two sensor pairs (1,3,5,7 and 2,4,6,8) odometry leads to large errors if both sensor pairs (2,4,6,8) are affected by bad contrast instead of only one pair (1,3,5,7). In case of sensor pair 3,7 suppressing the response of one of them is intolerable because rotation and translation cannot be separated and odometry leads to uncontrollable results. Therefore results for pair 3,7 have been omitted from Figure 11d,f. In general the more sensors are included, the safer the path-estimate. From a technical point of view eight sensors are convenient because synchronous reading of data from eight sensors by a microprocessor can be done by one-byte operations.

## 6. Discussion

In the case of superimposed self-rotation and -translation the ambiguity in the separation of rotation and translation poses a serious problem if only the limited FOV of a normal camera is available. The advantage of monitoring flow in an omni-directional FOV (like in insects) has been demonstrated [16,33,34]. The evaluation of self-motion from flow extracted from catadioptric systems [18–20] poses its own problems [33–35] and affords considerable computing power on the panoramic images. OMCs directly provide us with fast flow estimates along their viewing direction through their implemented dedicated DSP.

Since it is only necessary to properly combine a few widely distributed OF measurements for the evaluation of self-motion, we wanted to find out what can be done with only eight sensors for a ground moving vehicle. The ‘proper combination’ of OF results in various viewing directions simply means to apply a ‘matched filter’ to these results in order to find best estimates for the self-motion components. In our case the ‘unit’ responses mentioned in Chapter 3 which we tried to illustrate in Figures 2 and 5 represent such filters and their application to the sensor responses is shown in Equations (6) and (7) for the self-translation- and -rotation-components, respectively, of the carriage. Because the odometer registers only *increments* of path length and orientation between two samples, we expect increasing error accumulation the longer the track.

In Table 1 early results for a short pure translation of 80 cm and a rotation on the spot of 360° are presented. The error does not increase to more than about 0.2% in both cases.

Later the tracking camera was installed in order to record longer curved paths of the odometer head. On the curved trail of about 10 m with superimposed translation and rotation (see Figure 9) the error in the heading angle *α* accumulates to about 8° (Figure 10a), that of the traveled distance to about 7 cm (Figure 10b). The maximum distance between the positions gained from the odometer and the camera was 9 cm after about 820 cm of the track. From all our tests we conclude that under the favorite conditions of a well textured and illuminated flat ground the error of orientation as well as of position revealed by our odometer accumulates to no more than about 1% of angle or pathlength, respectively.

We would like to stress four more points :

(a) the response of mouse detectors is surprising linear for a wide range of flow velocities from slow motion up to the largest speed in our experiments. We never reached the maximum allowed speed of about 9 m/s (see Section 2) with hand controlled movements of the carriage within the 165 × 125 cm^2^ FOV of our camera at the ceiling. The calibration responses in Figure 5 have been recorded while pushing the carriage by hand which means large variations in traveling speed. Still the accumulated *X, Y*-responses of the sensors grew linearly with traveled distance and heading angle irrespective of the traveling- or angular speed profile. Note the very small slope of the accumulated *Y* response of the sensors in the case of yaw (lower panel of Figure 5). The small increase of the *Y*-response results from small deviations from the sensors' ideal orientation with respect to *X*-axes parallel to the ground plane. These deviations result in a small flow component in *Y* -direction during yaw. Even for these small flow components the accumulated *Y*-response deviates from a linear increase with yaw angle by a few counts irrespective of the yaw velocity. This means that our mouse sensors respond reliably down to very slow displacements of the pattern on their pixel array.
  The linearity of the response of mouse chips with respect to flow velocity is superior to the Reichardt detector and all its elaborated versions (e.g., [36]). The performance of the lens equipped mouse sensor seems to be also superior to other hardware based flow detectors such as the DVS sensor ([37]) (which has its merits in flow detection in an extended visual field) or the 2-pixel time of travel sensor ([38]), both with respect to velocity range and linearity. In particular, for the performance data of our mouse detector ADNS2620, we obtain much better results compared to those of the ADNS-9500 (Avago) shown in [38], maybe due to our attached optics (enlarged aperture, CAY046 lens).
(b) We did not investigate the influence of the amount of illumination of the scenery on the sensor recordings. But we saw no difference in sensor responses outdoor or indoor with an average room illumination. We used a lens with a numerical aperture of 0.4 which produces a relatively bright image compared to other applications using a lens with N = 0.2 (CAX100) [38,39]. If a lens is to be attached to the sensor, it is necessary to widen the stop provided by the factory in the cover of the sensor. All the lights from the lens should hit the light sensitive pixel area of the sensor. Mouse chips are intended to work on surfaces with a wide variety of reflectance factors. The built in adaptation of the shutter time to various illuminations of the pixel surface compensates for illumination changes.
(c) In order to detect flow induced by self-motion it is advantageous to look along directions which deviate as much as possible from the direction of self-translation or the axis of self-rotation. Because the translation induced flow is inversely proportional to the distance of seen contrast, ground moving agents should look directly down to the ground. But then they would not see any yaw (except the yaw axis is excentric to the sensor). A compromise which allows to see flow induced by yaw as well as by translation is to look at 45° to the ground. Then the flow induced by yaw is reduced only by a factor of $1/\sqrt{2}$ compared to a sensor with a horizontal optical axis. In the case of forwards translation the flow in a sensor looking down at 45° in forwards/backwards direction is reduced by a factor of 1/2 compared to flow in a vertically downwards looking sensor. A sensor looking sidewards and downwards under 45° sees a flow reduced only by a factor of $1/\sqrt{2}$ compared to a down looking sensor.
(d) Monitoring the *SQ*-response of the sensors allows one to exclude sensors that do not see good contrast. The more sensors are used the less the danger to find not enough good flow measurements for a safe self-motion estimate.

The advantages of motion detectors looking into a set of fixed selected directions over a camera are obvious :

(1) The sensors are light, cheap, and can be attached to various locations on the vehicle.
(2) Motion detection by mouse chips is at least 20 times faster than by a camera (1500 (*dX*, *dY*) samples/sec versus 60 frames/s). There is no need to wait for finishing a frame. In addition, flow needs not be extracted by some algorithm but is determined by a fast dedicated on-chip hardware.
(3) Motion detection can be done in parallel along as many lines of sight as sensors are used (simultaneous distributed flow extraction).
(4) Illumination problems are reduced because of the self adjusting exposure time of the chips to changing luminance of the environment.
(5) The focal length *f* of the lens attached to the sensor determines the FOV and the sensitivity to motion. A large *f* increases the sensitivity to motion (and decreases the FOV) but also increases the weight of the optics because *f*/*d* should not exceed a level of say 5 (depending on the luminance of the environment) in order to provide enough light on the sensor surface. The decreased depth of field with increased *f* does not play a critical role because some blur can be tolerated as long as the quality byte indicates a reliable intensity distribution on the sensor's surface.
(6) The arrangement of lines of sight of the sensors can be adjusted to the intended purpose (self-motion on the ground or in the air, obstacle avoidance, *etc.*). For self-motion estimates the angular distribution of viewing directions to contrast in the environment should be as wide as possible, preferably arranged in pairs of opposite viewing directions. For obstacle avoidance the motion sensitivity should be as large as possible and directed to nearly forwards directions.

Disadvantages are:

(1) Objects cannot be discriminated.
(2) Calibration may be a problem.
(3) Enough sensors must see contrast along their line of sight. But sensors that do not see enough contrast or respond irregularly may be excluded from motion estimate (because their quality byte indicates an unreliable intensity distribution of the sensor image) in order to make the estimate through the rest of the sensors reliable.

The advantages of OMCs on small unmanned aerial vehicles (SUAV) are also obvious. As already mentioned in the introduction a set of seven OMCs has been used by the LIS team of the EPFL (see the comprehensive PhD thesis of A. Beyeler [32] and [15] to construct a device called ‘opti pilot’ which controls the flight of a flying delta platform and allows to avoid large obstacles. Nevertheless the full potential of OMCs is not yet exploited, e.g., all three DOFs for rotation could quickly and easily be extracted from a set of eight sensors looking in opposite directions along the horizon and applying suitable matched filters to the OMC responses. A more difficult question is the extraction of true self-translation and distance to obstacles. For this purpose inspiration can be drawn from the observation of the side-to-side flight maneuvers of bees approaching a goal [40]. During these maneuvers the honeybees stabilize their head position against any rotation and thus avoid flow induced by self-rotation in their eyes. From the flow induced by self-translation they can judge absolute distances to contrast in forwards direction as long as they know their lateral speed. A first approach in this direction is reported by Adrien Briod ([39]).

## Figures and Tables

**Table 1.** Test results for head translation and rotation by hand.

**Translation**			**Rotation**		
	
*τ* [cm]	avg *τ̅* [cm]	std(*τ̅*) [cm]	*ρ* [deg]	avg *ρ̅* [deg]	std(*ρ̅*) [deg]
20	19.8495	0.1072	90	90.0205	0.4356
40	39.875	0.1064	180	180.3925	0.6167
60	59.8995	0.1425	270	271.1805	0.5264
80	79.9285	0.1710	360	360.664	0.8132
